# Patterns of surgical management of hollow organ injuries in severe trauma

**DOI:** 10.1007/s00068-026-03150-3

**Published:** 2026-03-17

**Authors:** Leonhard Andreas Schurr, Maria José de Schultz, Paul Kupke, Claudius Thiedemann, Till Kaltofen, Volker Alt, Hans Jürgen Schlitt, Daniel Popp

**Affiliations:** 1 Department of Surgery, University Medical Center Regensburg, Franz-Josef-Strauss-Allee 11, D-93053 Regensburg, Germany; 2 Department of Trauma Surgery, University Medical Center Regensburg, D-93053 Regensburg, Germany

**Keywords:** Abdominal trauma, Bowel injury, Damage control surgery, Decision making, Severely injured patients

## Abstract

In the setting of severe trauma, abdominal injuries are common and potentially life-threatening. Such injuries predominantly affect parenchymal organs, particularly the liver and spleen. Hollow organ injuries occur less frequently but nevertheless require timely and accurate diagnosis as well as appropriate surgical management to avoid severe complications, including peritonitis and sepsis. In daily clinical practice, different surgical strategies are employed for the management of hollow organ injuries. According to overall injury severity and abdominal injury patterns, treatment may involve either definitive single-stage surgery or staged procedures incorporating damage control principles. This study aimed to retrospectively evaluate the incidence and patterns of surgical management of hollow organ injuries in severely injured patients and to describe postoperative course and complications associated with different treatment strategies. Between 2006 and 2020, 1794 patients aged ≥ 16 years with an Injury Severity Score (ISS) ≥ 16 were treated at University Medical Center Regensburg, a level 1 trauma center. Among these, 57 patients sustained transmural hollow organ and/or mesenteric injuries requiring surgical intervention. Definitive surgical management during the initial operation was performed in 20 patients, whereas 37 patients underwent staged management with at least one planned secondary procedure. Patients managed with staged approaches exhibited higher overall injury severity and more complex abdominal injury patterns, including combined hollow organ and/or mesenteric injuries in 24.3%. Surgical treatment comprised bowel repair, resection with anastomosis, ostomy formation, or combinations of these techniques, carried out either during the initial operation or during planned subsequent procedures. No bowel leakage requiring revision surgery occurred following single-stage management. By contrast, bowel leakage necessitating unplanned reoperation was observed in 4 of 37 patients (10.8%) treated with staged approaches. In this retrospective single-center analysis, single-stage definitive surgical management was mainly observed in patients with moderate overall injury severity and less complex abdominal injury patterns, whereas staged strategies incorporating damage control principles were more frequently applied in patients with higher injury severity and complex abdominal injuries. These findings indicate that single-stage management may represent a feasible option in selected severely injured patients, while more complex injury patterns are more commonly managed using an individualized, staged surgical approach.

## Introduction

Abdominal injuries are a frequent component of severe trauma and predominantly involve parenchymal organs such as the liver and spleen. Hollow organ injuries are less common, with a reported prevalence of approximately 1 % in patients sustaining blunt trauma and up to 17 % in those with penetrating trauma [1]. Detecting such injuries during the initial assessment can be challenging [2]. Hemodynamically stable patients usually undergo imaging with computed tomography (CT). However, the diagnostic accuracy of CT in detecting hollow organ injuries remains controversial, as the sensitivity and specificity of certain radiological signs vary substantially [1–4]. CT-based scoring systems such as BIPS and RAPTOR have been proposed as useful tools for the assessment of emergency CT scans in patients with suspected hollow organ injuries [5, 6]. Nevertheless, a thorough physical examination remains indispensable, and the decision for surgical exploration should be based on a combination of clinical and radiological findings [7]. The presence of a seatbelt sign, for instance, should raise the suspicion of bowel injury [2, 8]. Among patients requiring trauma laparotomy who present with a seatbelt sign, small bowel injuries have been reported as the most common (58%), followed by large bowel (39%) and splenic injuries (39%) [8]. The presence of free fluid and other suggestive findings on CT, together with abdominal guarding, have been associated with earlier repair of bowel injuries, particularly in the absence of solid organ lacerations [9]. Diagnostic laparoscopy represents a valuable tool in hemodynamically stable patients when both clinical assessment and radiological imaging fail to provide a definitive diagnosis. [1]. Surgical management can then be carried out via conversion to laparotomy or, depending the surgeon’s experience, laparoscopically [1, 10]. Options in treating hollow organ injuries include repair by suture, resection with or without anastomosis as well as creation of an ostomy [11]. Definitive management of the injury can either be performed during the initial operation or can be delayed with the initial operation aiming for control of bleeding and contamination [12]. The latter approach is widely known as Damage Control Surgery (DCS). Primary repair by direct suture of a bowel injury involving less than 50% of the circumference and preserved perfusion is feasible [1]. In cases requiring bowel resection, an anastomosis may be performed either primarily or in a delayed fashion, although delayed anastomoses have been associated with increased leakage rates [13, 14]. Furthermore, colonic anastomoses show higher leak rates the more distally they are located, reaching up to 50% in the left colon [15]. An open abdomen and delayed fascial closure are additional factors that can increase the risk of leakage, particularly in colonic anastomoses [14, 16, 17]. Ostomies, either loop or end stomas, represent the safest option in managing hollow organ injuries. According to the current literature, they should be considered in the presence of multiple colonic anastomoses or other high-risk situations [1].The aim of this study was to retrospectively analyze incidence, surgical management and clinical course of hollow organ injuries in severely injured patients with an ISS of 16 or higher. Rates of unplanned reoperations and bowel leakage were examined and compared between groups with different treatment strategies to assess whether single-stage management of hollow organ injuries is feasible in the context of severely injured patients, as opposed to strategies including a programmed second look operation.

## Patients and methods

All severely injured patients treated at our level 1 trauma center between 2006 and 2020 were screened. Inclusion criteria were an ISS of ≥ 16 as well as age of ≥ 16. Ethical approval was obtained by the Ethics Committee of the University of Regensburg (25-4276-104). Primary data collection was performed by study assistants independently of the treatment team as part of a prospective single-center trauma database. The data are also being entered into a national registry (TraumaRegister, DGU®). More specific parameters were filled in by the authors retrospectively. All patients (n=1794) were primarily evaluated regarding the presence of hollow organ injuries and/or mesenteric injuries as well as the necessity for surgical intervention. Both blunt and penetrating mechanisms were included. 57 patients who met the above criteria could be identified. The patients were then divided into the following groups to compare different surgical treatment strategies:

Group 1: Patients with definitive surgical management at the initial operation (n=20).

Group 2: Patients with delayed definitive management (n=37).

For more detailed analysis Group 2 was further split up in three subgroups:

Group 2a: Patients with primarily definitive management regarding the bowel, but who underwent a programmed second look, mostly due to concomitant abdominal injuries (n=10).

Group 2b: Patients with initial DCS (resection and blind closure of both ends) and delayed definitive management regarding the bowel (n=19).

Group 2c: Patients who deceased prior to definitive management regarding the bowel (n=8).

Data collection included demographic parameters, ISS, pre- and in-hospital management, abdominal injury patterns, clinical course (including duration of ventilation, length of stay on ICU and overall hospitalization) and short-term outcome (30-day mortality). Specific information regarding the hollow organ as well as mesenteric injuries and their operative management was obtained. All groups were analyzed in a descriptive fashion. Moreover we compared the groups regarding their postoperative course and complication rates. All statistical analysis was conducted using SPSS version 25 software (SPSS Inc., Chicago IL, USA). Binary or nominal target variables were investigated using Chi-Square-Test and Fisher’s exact test. Kolmogorov–Smirnov test was used to test for normal distribution. Mean values among the groups were compared by unpaired samples t-test. Mann–Whitney-U-test was performed to compare median values. P-values of < 0.05 were considered statistically significant.

## Results

During the study period, 1794 patients with an ISS of ≥ 16 as well as age of ≥ 16 were treated at our institution. 57 cases (3.2%) revealed hollow organ injuries and/or mesenteric injuries and received surgical treatment. These patients were divided into groups, as mentioned earlier. The following table contains demographic data as well as parameters describing their overall clinical course Table 1.

**Table 1:** Demographic and clinical parameters

	Overall (n=57)	Group 1 (n=20)	Group 2 (n=37)	Group 2a (n=10)	Group 2b (n=19)	Group 2c (n=8)
Median age (range)	43 (16-89)	32 (16–87)	45 (17–89)	44 (20–89)	46 (17–75)	51 (24–81)
Sex ratio m/f	41/16 (m=72 %)	15/5 (m=75%)	26/11 (m=70%)	6/4 (m=60%)	12/7 (m=63%)	8/0 (m=100%)
Median ISS (range)	34 (17-75)	25^1^ (17-57)	36^1^ (17-75)	40 (17-59)	34 (24-75)	38 (25-50)
Prehospital intubation	40 (70%)	10^2^ (50%)	30^2^ (81%)	8 (80%)	15 (79%)	7 (88%)
Median SBP on adm. in mmHg	110 (90-128.5)	121^3^ (96.3-140)	103^3^ (83.3-125.3)	96.5 (86.5-126.8)	104 (80-124)	115 (100-125)
Median nr of pRBCs	4 (0-9.8)	0^4^ (0-3.8)	5.5^4^ (2-16.8)	3.5 (0-7)	7 (2-20)	11.5 (4-22.3)
Median nr of FFPs	6 (0-20)	0^5^ (0-0)	12^5^ (3.8-25.5)	6 (0-14.8)	12.5 (6-24.5)	25 (3-34.8)
Median ICU length of stay	12d (4-24)	9.5d (3-21.5)	14d (5.5-25)	12d (9.8-21.8)	18d (12-35)	2.5d (1-4)
Median duration of ventilation	4d (1-11.5)	1d^6^ (0-11)	5d^6^ (2-12)	8d (2-9.3)	6d (3-24)	2.5d (1-4)
Median total hospital stay	23d (11.5-37)	22.5d (12.3-34)	24d (11-38)	22.5d (16.3-29.8)	34d (21-59)	2.5d (1-4.8)
30 day-mortality	10 (18%)	0^7^ (0%)	10^7^ (27%)	2 (20%)	0 (0%)	8 (100%)

SBP on adm.  = systolic blood pressure on admission, nr of pRBCs = number of packed red blood cell units during initial resuscitation (transfused in trauma bay and initial operation), nr of FFPs = number of fresh frozen plasma units during initial resuscitation;  p^1^=0.002, p^2^=0.014, p^3^=0.007, p^4^<0.001, p^5^<0.001, p^6^=0.017, p^7^=0.01

**Fig. 1:**
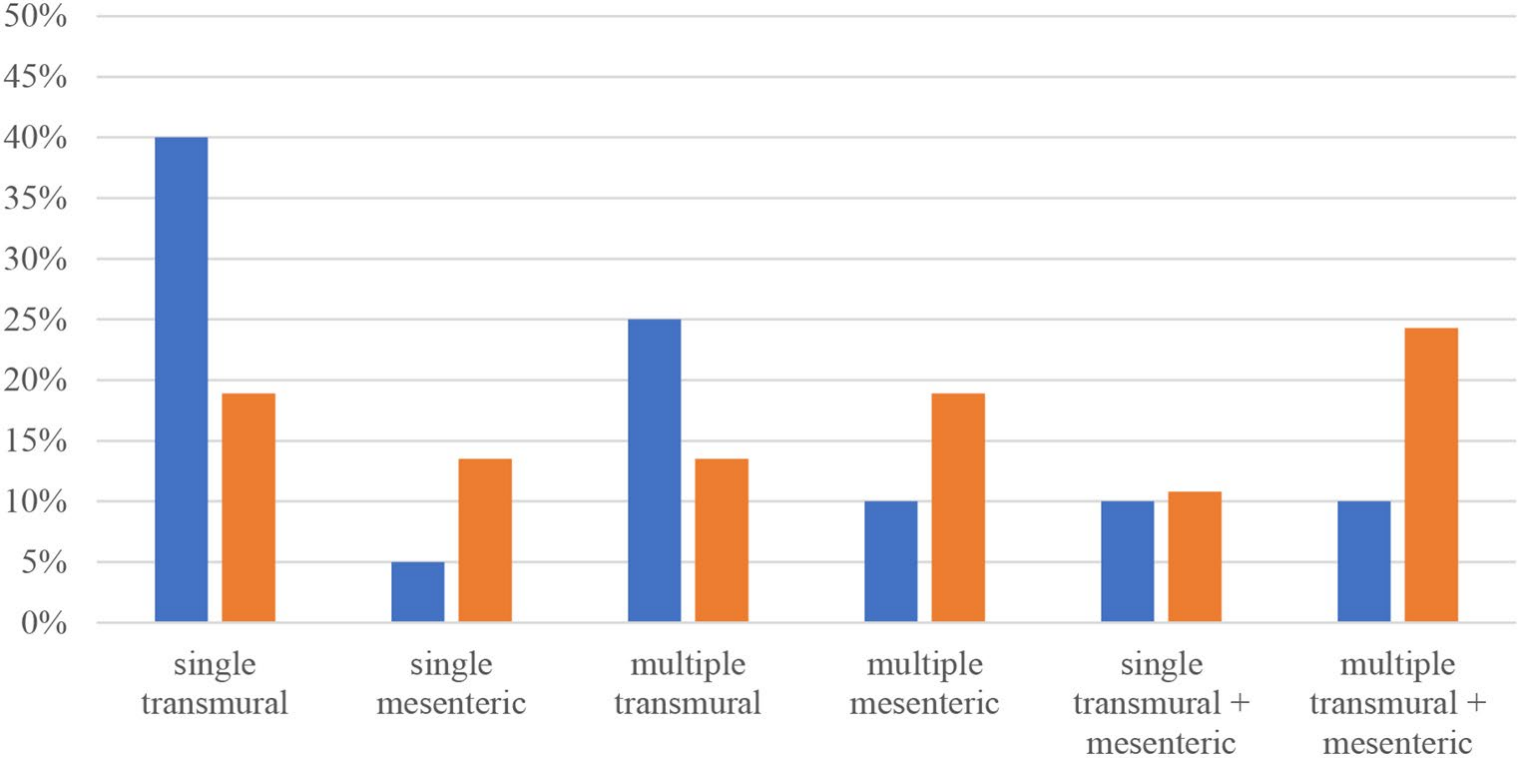
Types of injuries in groups 1 (blue) and 2 (orange)

**Fig. 2:**
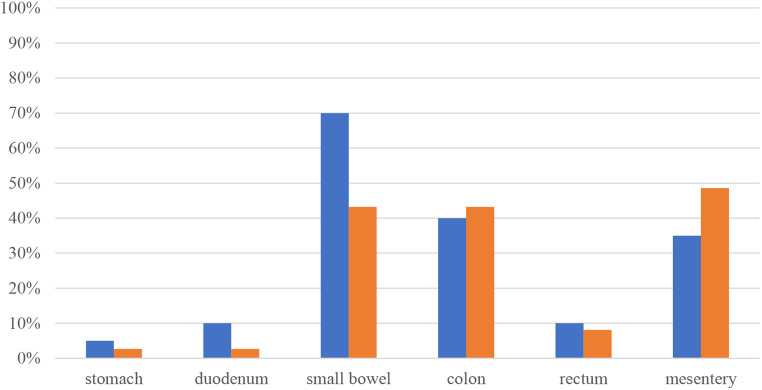
Injury sites in groups 1 (blue) and 2 (orange)

**Fig. 3:**
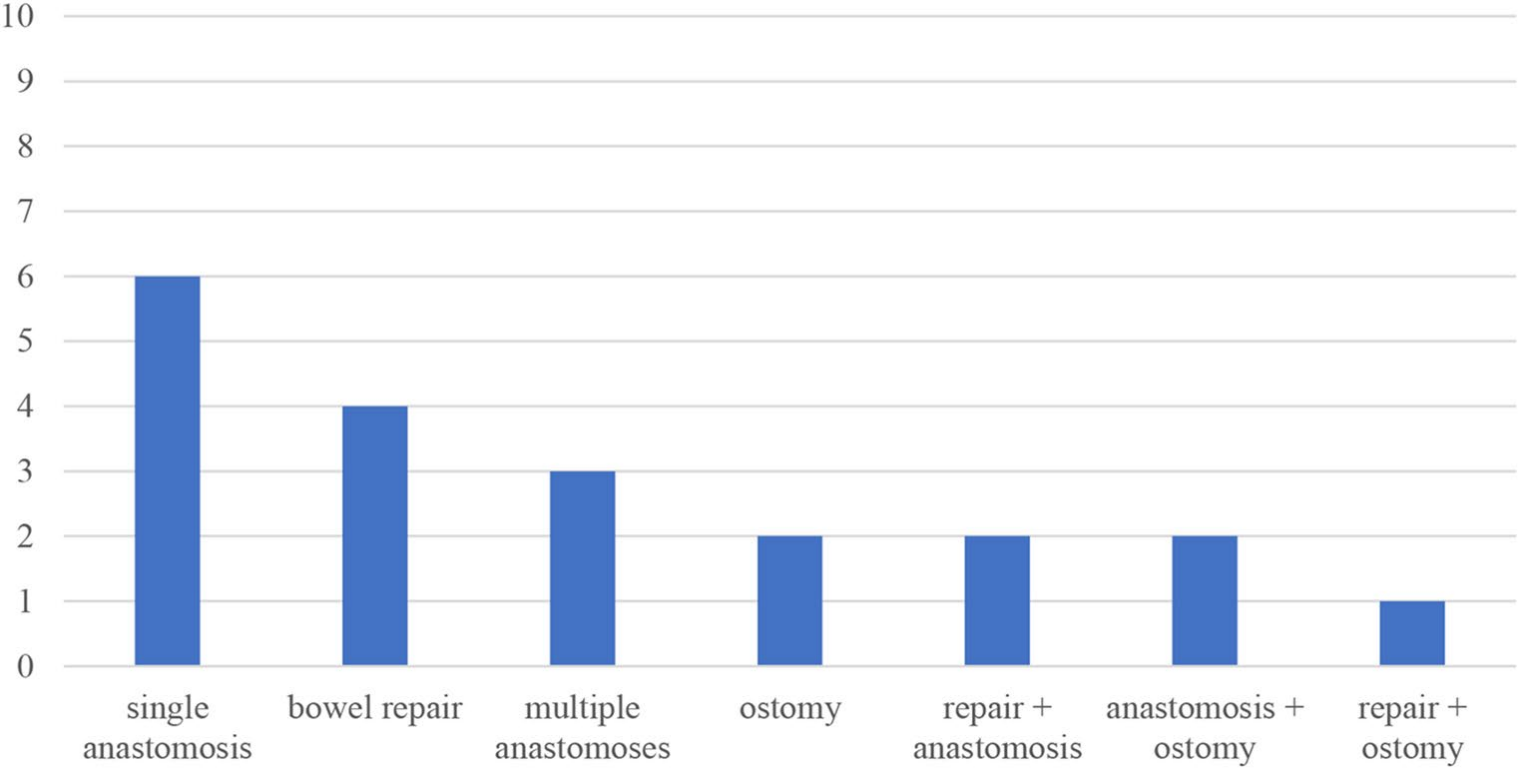
Surgical procedures in group 1

**Fig. 4:**
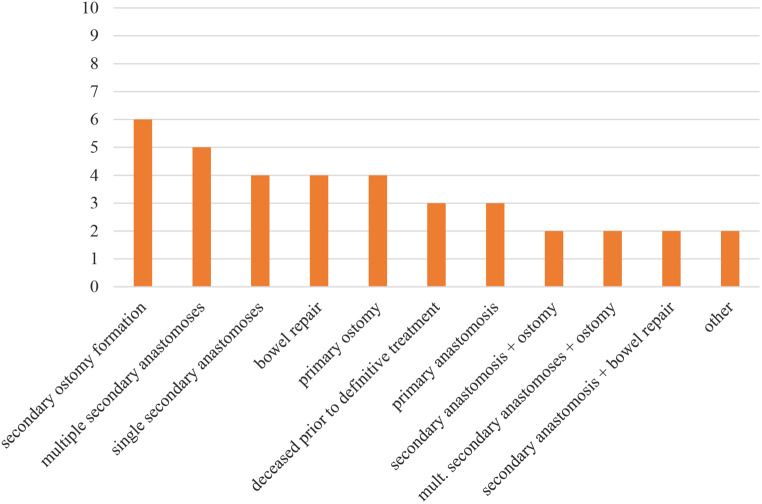
Surgical procedures in group 2

No significant differences in age or sex ratio could be detected, all groups showed similar male predominance. Median ISS was significantly higher in group 2 compared to group 1 (p=0.002). Furthermore, patients in group 2 had undergone prehospital intubation significantly more often (p=0.014) and presented with lower systolic BP on admission (p=0.007). During initial resuscitation in the trauma bay and the initial operation patients in group 1 had significantly lower transfusion requirements regarding pRBCs as well as FFPs (p<0.001, respectively). Postoperatively patients in group 2 required longer ventilation times (p=0.017). 30 day-mortality in group 1 was 0%, significantly less than 27% in group 2 (p=0.01).Overall, 50 patients (87.7%) sustained blunt trauma, predominantly due to motor vehicle accidents. There was no significant difference between groups 1 and 2 regarding the trauma mechanism (80% vs. 91.9%, p=0.187). A total of 51 patients (89.5%) were directly transferred to the operating room after initial treatment in the trauma bay, with no significant difference between groups 1 and 2 (85.0% vs. 91.9%, p=0.35). The mean time from arrival to the start of abdominal surgery was 89.7 ± 33.3 minutes (range 38–162) in group 1 and 60.4 ± 34.1 minutes (range 10–150) in group 2, which was significantly shorter in group 2 (p=0.025). The types of injuries found intraoperatively are depicted in the following Fig. 1–4.

Patients in group 1 predominantly suffered from single or, second most frequently, multiple transmural injuries that could be managed by a single operation. Patients in group 2, who require at least one second look, most commonly showed a pattern of multiple mesenteric as well as transmural bowel injuries. Abbreviated Injury Scale (AIS) severity levels ranged from 2 to 5, with an overall mean value of 3.75 and no significant differences between the groups.

The following figure depicts percentages of patients in each group that revealed injuries to the corresponding organ. Combination injuries commonly occurred, as mentioned above. Patients in group 1 were mostly treated for small bowel injuries (70%), in group 2 mesenteric injuries (48.6%) were followed closely by damaged small bowel (43.2%) and colon (43.2%).

Surgical management of the patients’ injuries was analyzed and categorized in bowel repair by direct suture, anastomosis, formation of an ostomy or a combination of different procedures. As the following figure shows, patients with early total care (group 1) received a single anastomosis as the most common procedure. After that, direct bowel suture, multiple anastomoses, formation of an ostomy and combination of different techniques were performed in descending frequency.

Patients in group 2 received a variety of different procedures, as depicted in the figure above. Among all solutions the secondary formation of an ostomy was most frequently chosen, followed by multiple secondary anastomoses.

Groups 1 and 2 were compared regarding the occurrence of unplanned reoperations. Revision-rates were similar in both groups: 6 patients (30%) in group 1 and 13 patients (35.1%) in group 2 were taken back to the operating room, the difference was not statistically significant (p=0.417). 

The rate of postoperative bowel leakage was investigated in both groups. None of the cases in group 1 and 4 patients (10.8%) in group 2 developed a leak, which was significant (p=0.016). 2 leaks corresponded to multiple insufficient anastomoses, one was a single leaking anastomosis and in one case multiple anastomosis as well as an ostomy failed to heal in a particularly complex case. 3 out of the 4 cases occurred in subgroup 2b, meaning that definitive treatment was delayed after initial DCS. 

Other frequent reasons for revision surgery besides bowel leakage were surgical site infections, fascial dehiscence, hematoma or abdominal compartment syndrome.

## Discussion

Management of severely injured patients represents a multidisciplinary challenge for all medical professionals involved. After initial treatment in the prehospital phase as well as in the emergency department, surgical interventions are often necessary. The question of how to deal with hollow organ injuries cannot be answered in a simple way. The principle of DCS was established to reduce mortality in severe trauma by quickly controlling bleeding and peritoneal contamination during initial exploration in order to allow for earlier transfer to intensive care units for stabilization. Definitive management of bowel injuries then has to be carried out during a programmed second look. On the other hand, as mentioned earlier, delayed fascial closure and delayed anastomosis are risk factors for bowel leakage and thereby for increased morbidity [13, 14, 16, 17]. These facts have to be weighed against each other carefully. To analyze surgical management of hollow organ injuries among severely injured patients we categorized our patients into cases that received definitive treatment at their initial operation (group 1) and those, who underwent programmed reoperations (group 2). It was not surprising that patients in group 1 presented with a significantly lower ISS, accompanied by lower rates of prehospital intubation and higher systolic blood pressure on admission. Transfusion requirements during initial resuscitation were also lower in this group. Together, these findings indicate a lower overall trauma severity. Patients in group 2 were transferred to the operating room significantly faster than those in group 1, most likely reflecting a greater surgical urgency. No significant delay in diagnosing hollow organ injuries was observed in either group. Treatment in group 1 was associated with significantly shorter duration of ventilation and no 30-day mortality, which was significantly lower than the 27% observed in group 2. This already indicates that therapeutic decision making towards primary definitive surgical care did not lead to higher mortality: None of these 20 patients seems to have needed DCS to survive his or her severe trauma. The patients’ abdominal injury patterns were analyzed further. Patients in group 1 most frequently presented with single transmural small bowel injuries and were managed by a single anastomosis. In group 2 complex combination injuries of transmural bowel lesions and mesenteric tears were the most common pattern, mostly receiving a delayed ostomy. To assess whether decision-making towards a single-stage strategy was justified, we looked at rates of unplanned reoperations. No significant differences could be found, indicating that primary definitive care did not result in higher surgical complication rates. The probably most important finding was that no bowel leak occurred among any of the 20 cases in group 1. The 4 leakages (10.8%) in group 2 were significantly more, not surprising due to the findings of previous studies, as mentioned above. Our findings underline that even in a collective of severely injured patients, definitive surgical care at the primary operation can be carried out safely with good results, if abdominal and overall injury patterns are not excessively complex. Without any doubt DCS plays an important role in managing those cases with higher severity. These patients would be exposed to unnecessary risk by prolonged surgical procedures in the acute phase. In surgical decision-making, each case still has to be looked at individually. Our findings are limited by a relatively small patient collective and the design as retrospective single-center investigation. Further investigations with larger patient collectives are needed to precisely describe possible cut-off parameters for DCS in bowel injuries. 

## Conclusion

Hollow organ injuries in severely injured patients are rare. Surgical management may be carried out either as primary definitive care or via DCS with at least one planned secondary operation. Primary definitive care was observed to be feasible and safe in patients with moderate overall injury severity, particularly when only the small bowel was involved, and could often be managed with a single anastomosis. In patients with complex abdominal injury patterns or higher overall injury severity, an individualized approach incorporating DCS was more frequently applied.

## Statements

## Data Availability

The data that support the findings of this study are derived from a prospective single-center trauma database at the University Medical Center Regensburg and are also entered into the national TraumaRegister (DGU^®^). Due to patient confidentiality and institutional regulations, individual patient data are not publicly available. Aggregated data underlying the results reported in this manuscript can be made available from the corresponding author upon reasonable request and with permission from the University Medical Center Regensburg.
